# Kinetic modelling of [⁶⁸Ga]Ga-FAPI-46 PET in pancreaticobiliary lesions: distinguishing cancer from pancreatitis

**DOI:** 10.1007/s00259-026-07906-2

**Published:** 2026-05-06

**Authors:** Ted Nilsson, Pawel Rasinski, Ernesto Sparrelid, Antonios Tzortzakakis, Thuy A Tran, Örjan Smedby, Rimma Axelsson, Mark Lubberink, Maria Holstensson

**Affiliations:** 1 Department of Clinical Science, Intervention and Technology, Karolinska Institutet, Stockholm, Sweden; 2 Department of Nuclear Medicine and Medical Physics, Karolinska University Hospital, Huddinge, Sweden; 3 Theranostics Trial Center, Karolinska University Hospital, Huddinge, Sweden; 4 Department of Upper Abdominal Diseases, Karolinska University Hospital, Huddinge, Sweden; 5 Department of Oncology-Pathology, Karolinska Institutet, Stockholm, Sweden; 6 Department of Radiopharmacy, Karolinska University Hospital, Stockholm, Sweden; 7 Department of Biomedical Engineering and Health Systems, KTH Royal Institute of Technology, Huddinge, Sweden; 8 Department of Molecular Medicine and Surgery, Karolinska Institutet, Stockholm, Sweden; 9 Molecular Imaging and medical physics, Department of Surgical Sciences, Uppsala University, Uppsala, Sweden

**Keywords:** Kinetic modelling, Dynamic PET, [⁶⁸Ga]Ga-FAPI-46, Pancreatic cancer

## Abstract

**Purpose:**

Fibroblast activation protein (FAP)–targeted PET using [⁶⁸Ga]Ga-FAPI-46 visualizes fibroblasts abundant in pancreatic cancer (PC) but also present in pancreatitis, complicating interpretation of static images. Dynamic imaging and kinetic modeling may provide additional insight, but their diagnostic value remains unclear. This study evaluated whether kinetic parameters from dynamic [⁶⁸Ga]Ga-FAPI-46 PET can differentiate PC from pancreatitis and their relationship with standardized uptake value (SUV) and tumor-to-blood ratio (TBR).

**Methods:**

Sixty-one patients with suspected pancreaticobiliary cancer underwent a 45-min dynamic [⁶⁸Ga]Ga-FAPI-46 PET scan, followed by static scans at 60 and 180 min. Time–activity curves were generated for 51 malignant and 53 benign lesions. Compartment models and Logan analysis yielded kinetic parameters (K_1_, k_2_, k_3_, k_4_, V_T_, V_NS_, V_S_). SUV and TBR were correlated with V_T_, and group comparisons and ROC analyses assessed discriminatory performance.

**Results:**

Reversible models best described the tracer kinetics. V_T_ and V_S_ were significantly higher in PC than pancreatitis, and k_2_ and k_4_ were lower, indicating higher [⁶⁸Ga]Ga-FAPI-46 binding respectively slower washout in malignant lesions. SUV correlated strongly with V_T_ (*r* ≥ 0.784)​, and TBR showed very strong correlations (*r* ≥ 0.902)​ for the 0–60 min interval, with strong correlations observed across all models and time points. ROC analyses demonstrated comparable differentiation between V_T_, SUV_max_, and TBR_max_.

**Conclusions:**

Kinetic parameters showed strong correlations with simplified methods and similar ability to differentiate PC from pancreatitis. SUV and TBR measures thus represent practical alternatives to kinetic modelling for lesion characterization.

ClinicalTrials.gov ID: NCT05172310

**Supplementary Information:**

The online version contains supplementary material available at 10.1007/s00259-026-07906-2.

## Introduction

Increasing attention has been paid to fibroblast activation protein (FAP) as a molecular imaging target in oncology, owing to its high expression of cancer-associated fibroblasts (CAFs) in approximately 90% of epithelial carcinomas [1]. Pancreatic cancer (PC) in particular, is characterized by a dense desmoplastic stroma, created by resident mesenchymal stellate cells that differentiate into CAFs [2, 3]. The current best practice imaging method for diagnosing and staging PC is contrast-enhanced computed tomography (CECT) supplemented by magnetic resonance imaging (MRI) in equivocal cases [4, 5]. However, distinguishing between concomitant PC and pancreatitis with CECT and MRI is difficult [6, 7], complicating both diagnosis and treatment planning. Patients with suspected PC detected at an early stage are often referred for pancreatectom**y.** A well-recognized clinical concern is that inflammatory pancreatic lesions may mimic malignancy, resulting in unnecessary surgery in patients without cancer [8, 9]. Thus, it is of great importance to find diagnostic tools that can improve the differentiation between malignant and benign lesions, thereby improving treatment selection and minimizing the number of unnecessary surgeries [9, 10]. Fibroblast activation protein inhibitors (FAPI)-based tracers target CAFs abundant in pancreatic tumors, and they hold promise for distinguishing malignant from benign lesions. Several studies have demonstrated the diagnostic potential of Positron Emission Tomography (PET) using radiolabeled FAPI tracers in PC [11–16]. However, since CAFs are also present in benign conditions such as pancreatitis and fibrosis [17, 18], differentiating concomitant malignant and benign lesions on static FAPI PET images can be challenging. Kinetic modelling of dynamic [⁶⁸Ga]Ga-FAPI-46 PET may help resolve this overlap by providing biologically relevant information beyond static measurements such as e.g. receptor binding and radiotracer delivery and clearance, thereby offering insights beyond static imaging [19, 20]. FAP expression can vary between PC and pancreatitis [21], and if the kinetic behavior differs between the lesion types, dynamic imaging and kinetic modelling could improve differentiation. Kinetic modelling of FAPI in gastric and pancreatic lesions has been studied in several studies in small cohorts [22–27]. In published studies so far, two different FAPI-ligands have been used: [⁶⁸Ga]Ga-FAPI-04, and [⁶⁸Ga]Ga-FAPI-46. Both FAPI ligands exhibit reversible binding and simplified methods such as Standardized uptake value (SUV) and tumor-to-blood ratio (TBR) have shown good correlation with the total volume of distribution (V_T_) [23, 27]. However, these studies involved small, heterogeneous cancer cohorts with limited histopathological verifications, especially in benign lesions with high concentration of FAPI-tracer.

Despite the growing interest in FAPI-PET, the potential of kinetic modelling to improve differentiation between PC and pancreatitis remains poorly understood, highlighting an important knowledge gap. Finding significant kinetic differences between PC and benign lesions could enhance diagnostic accuracy and potentially support appropriate treatment selection for patients. The primary objective of this study was to assess whether kinetic modelling parameters derived from [⁶⁸Ga]Ga-FAPI-46 PET can differentiate between PC and pancreatitis in a cohort of patients with suspected pancreatic malignancies scheduled for surgery A secondary objective was to evaluate how relevant kinetic parameters relate to simplified methods, and to explore whether kinetic modelling provides superior information for differentiation compared to SUV and TBR.

## Materials and methods

### Study design and patient cohort

Patients scheduled for surgery due to suspected PC or other periampullary cancer were enrolled in an ongoing prospective imaging clinical trial (NCT05172310; EUCT-2024-514967-25-00), in which they subsequently underwent [⁶⁸Ga]Ga-FAPI-46 PET/CT imaging at Karolinska University Hospital. Imaging was conducted within two weeks before surgery, during a period from 2021 to 2025. Histopathological findings obtained after surgery were used as the reference standard. The study complied with the Declaration of Helsinki and was approved by the Swedish Ethical Review Authority, and the Swedish Medical Products Agency. All participants provided written informed consent.

## Radiopharmaceuticals and imaging protocol

The [⁶⁸Ga]Ga-FAPI-46 precursor was obtained from SOFIE Biosciences (*Dulles*,* VA*,* USA*). Good manufacturing practice compliant automated radiosynthesis of [⁶⁸Ga]Ga-FAPI-46 was performed at the Karolinska radiopharmacy [28]. Prior to administration of the radiotracer a low-dose native CT was performed for attenuation correction. The mean administered [⁶⁸Ga]Ga-FAPI-46 activity was 203 ± 58 MBq. Dynamic PET/CT acquisition was initiated simultaneously with radiotracer administration and continued for 45-minute in list-mode using either a Siemens Biograph mCT PET/CT scanner (*Siemens Healthineers*,* Erlangen*,* Germany*) or a GE Discovery MI PET/CT scanner (*GE Healthcare*,* Waukesha*,* WI*,* USA*). The axial field-of-view was 21.6 respectively 20.0 cm. A single bed position, centered over the suspected primary lesion was used for the dynamic acquisition. The list-mode data were reconstructed into 33 frames (1 × 10s, 8 × 5s, 4 × 10s, 2 × 15s, 3 × 20s, 4 × 30s, 5 × 60s, 5 × 300s, 1 × 600s). Whole-body PET was performed 60 min post injection from skull to mid-thigh, preceded by a native CT for attenuation correction. PET data were acquired in step-and-shoot mode with 4 min per bed position. A diagnostic CECT was subsequently performed. Patients underwent an additional PET scan 180 min post-injection, acquired over a single bed position centered on the suspected primary lesion, with an extended acquisition time of 14 min to compensate for radiotracer decay. PET images were reconstructed using ordered-subset expectation maximization incorporating time-of-flight and resolution recovery, including all appropriate corrections, with scanner-specific parameters identical to those previously described [29]. Reconstruction harmonization was verified using a NEMA phantom to ensure comparable SUV and contrast across systems.

### Data analysis

PET/CT images from the 60- and 180-min post injection time points were co-registered in the software PMOD (*version 4.04; PMOD Technologies Ltd.*,* Zurich*,* Switzerland*) to the dynamic dataset. Volumes of interest (VOIs) were delineated in PMOD on the last frame of the dynamic PET acquisition, using a 40% isocontour of SUV_max_ by authors PR and TN in consensus. The co-registered CECT was used to support the delineation. VOIs with a volume < 1.5 cm³ were excluded from further analysis because of their higher sensitivity to motion. An image-derived input function (IDIF) was generated by placing a circular region of interest with a diameter of 10 mm, in ten contiguous slices in the descending aorta. The IDIF-VOI and lesion-VOIs were projected onto each of the 33 frames of the dynamic PET dataset to generate time activity curves (TACs). VOIs from the dynamic images were copied to the late timepoints to ensure intra-individual spatial consistency. Measurements from the 60- and 180-min timepoints were decay-corrected to the time of administration and appended to the TACs of the dynamic PET acquisition. SUV, normalized to body weight, was measured for each time point along with the TBR defined as the ratio of activity concentration in lesion to whole-blood activity concentration measured in the IDIF. For both SUV and TBR measurements, maximum, peak, and mean values were analyzed, resulting in SUV_max_, SUV_peak_, and SUV_mean_, respectively TBR_max_, TBR_peak_, and TBR_mean_.

## Tracer kinetic modelling

All pharmacokinetic modelling was performed in PMOD. Three compartment models were evaluated: the single-tissue-compartment model (1T2k), as well as the reversible (2T4k) and irreversible (2T3k) two-tissue-compartment models [20]. All models included an additional fit parameter for blood volume fraction (V_B_). Patlak and Logan plots were also applied to all lesions in the study [30, 31]. The nomenclature of kinetic rate constants (K_1_, k_2_, k_3_, k_4_) and macroparameters follows the consensus definitions proposed by Innis et al. [32]. For the 1T2k model the total volume of distribution V_T_ was calculated as $\:{V}_{T}=\frac{{K}_{1}}{{k}_{2}}\:$. For the 2T4k model V_T_ was calculated as $\:{\mathrm{V}}_{\mathrm{T}}=\frac{{\mathrm{K}}_{1}}{{\mathrm{k}}_{2}}\times\:(1+\frac{{\mathrm{k}}_{3}}{{\mathrm{k}}_{4}})$, representing the ratio of radiotracer in tissue to that of plasma at dynamic equilibrium. V_T_ is the sum of non-specific binding $\:{V}_{ND}=\frac{{K}_{1}}{{k}_{2}}\:$, and the specific binding $\:{V}_{S}=\frac{{K}_{1}\times\:{k}_{3}}{{k}_{2}\times\:{k}_{4}}$. For the 2T3k-model the net influx rate (K_i_) was calculated, representing the rate of irreversible radiotracer accumulation in FAP expressing tissue and is calculated as $\:{\mathrm{K}}_{\mathrm{i}}=\frac{{\mathrm{K}}_{1}\times\:\:{\mathrm{k}}_{3}}{{\mathrm{k}}_{2}+{\mathrm{k}}_{3}}$. Parameters with relative standard error > 25% were excluded to ensure reliable estimates.

### Statistical analysis

The Akaike information criterion (AIC) was used as a goodness-of-fit measure to determine the preferred compartment model [33]. Pearson correlation coefficient (R_P_) was used to determine the correlation between SUV, TBR, and parameters derived from kinetic modelling. Comparisons between malignant and benign lesion were made with a mixed linear model treating malignancy as a fixed effect and patient identity as a random effect. A p-value less than 0.05 was considered statistically significant. To correct for multiple comparisons, the Benjamini-Hochberg procedure with a false discovery rate of 0.05 was applied to p-values belonging to the same kinetic model. To quantify effect size, the marginal R² was calculated, representing the proportion of variance explained by the fixed effects of the statistical model, i.e., lesion malignancy [34]. Considering the assumptions of the linear model, the normality of residuals was tested with the Shapiro-Wilks test, and an alternative analysis was made with a rank-transformed version of each dependent variable, as recommended by Conover [35]. Receiver operating characteristics (ROC) analysis was used to compare the area under the curve (AUC) for differentiating malignant and benign lesions. The statistical analyses were performed in SPSS Statistics (version 29; IBM).

## Results

### Patients

Sixty-one patients with a total of 104 lesions were included in the study. Of these, 51 lesions were confirmed as malignant and 53 as pancreatitis based on histopathological analysis. Thirty-eight patients had both malignant lesions and chronic pancreatitis. In five patients’ histopathological confirmation was not available since pancreatectomy was not performed (four unresectable cases and one gallbladder cancer); these were diagnosed as pancreatitis based on clinical assessment and imaging findings. Malignant lesions included pancreatic cancer (*n* = 45), cholangiocarcinoma (*n* = 4), ampullary cancer (*n* = 1), and duodenal cancer (*n* = 1). Clinical characteristics of the patients are presented in Table 1. All included patients had dynamic imaging, and a static scan at 60 min post-injection. Thirty-three patients also had imaging at 180 min post-injection.

**Table 1:** Patient characteristics (*n*=61)

Characteristics	Data
Age (years), median (range)	70 (45 – 85)
Sex (n)	
Male	36
Female	25
Weight (kg), median (range)	74 (52 – 116)
Injected activity (MBq), mean (range)	203 (102 – 312)
Malignant lesion type (n)aw	
Pancreatic cancer	45
Cholangiocarcinoma	4
Ampullary cancer	1
Duodenal cancer	1
Benign lesion type (n)	
Chronic pancreatitis	53

## Tracer kinetic analysis

**Fig. 1:**
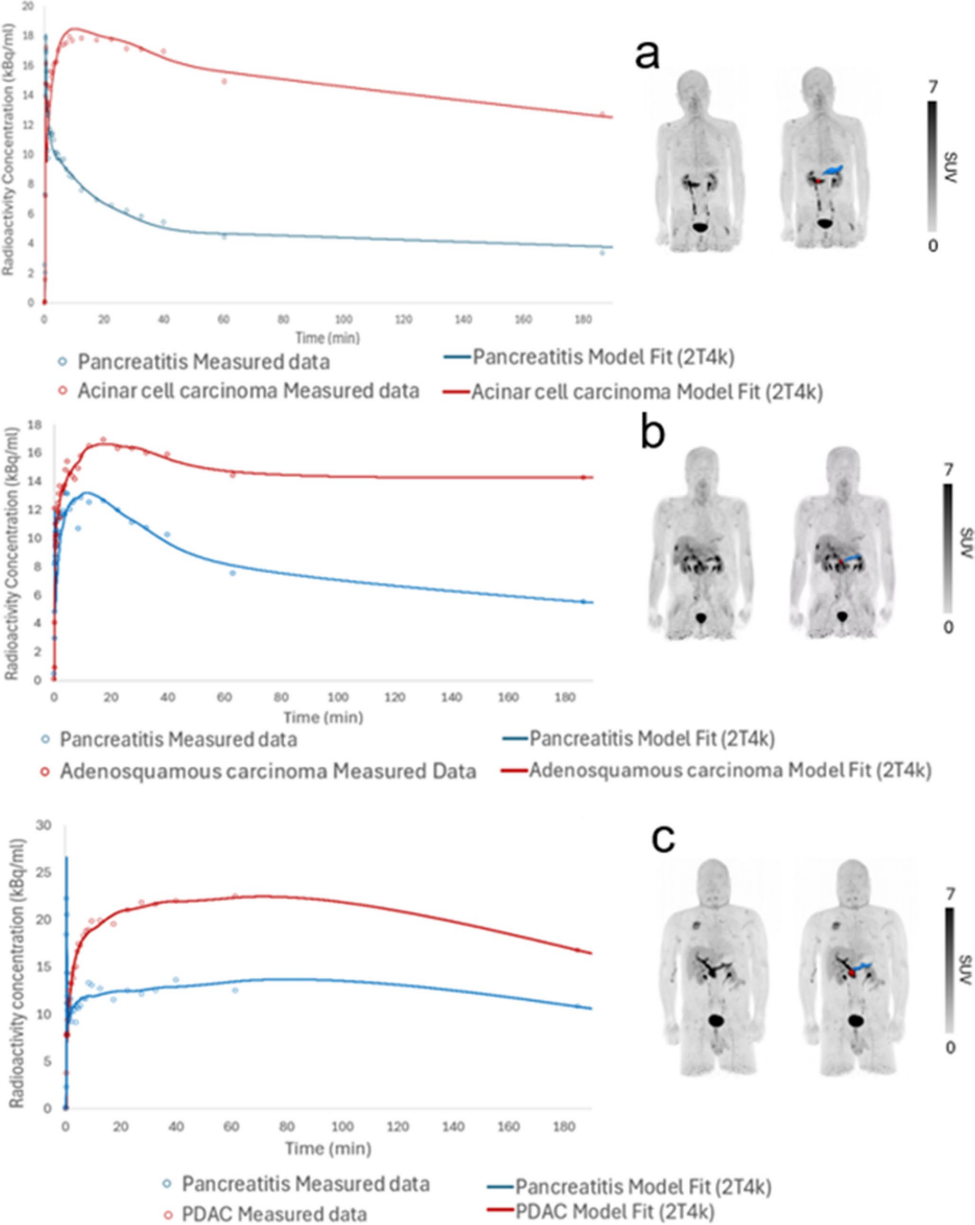
Time activity curves and corresponding 2T4k-model fits from 0–180 min in three patients with concomitant malignant and benign pancreatic lesions. (**a**) Acinar cell carcinoma with chronic pancreatitis. (**b**) Adenosquamous carcinoma with chronic pancreatitis. (**c**) Pancreatic ductal adenocarcinoma (PDAC) with chronic pancreatitis

**Fig. 2:**
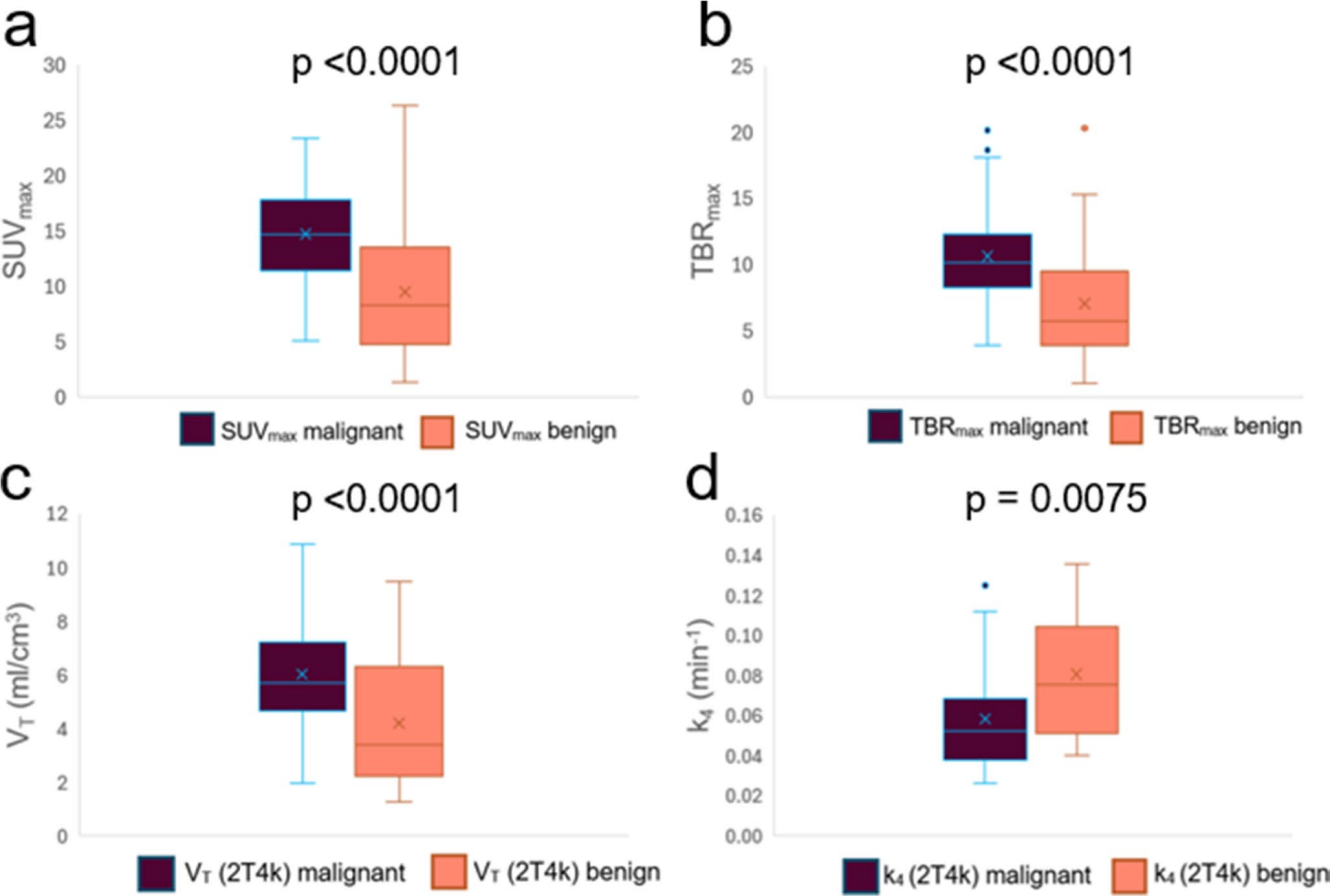
Boxplots illustrating SUV_max_ (**a**), TBR_max_ (**b**), V_T_ estimated using the 2T4k-model (**c**), and k_4_ from the 2T4k-model (**d**) in malignant and benign lesions from the 0–60 min interval

Figure 1 shows TACs and fitted 2T4k model curves for three patients with concomitant PC (acinar cell carcinoma, adenosquamous carcinoma, and pancreatic ductal adenocarcinoma) and chronic pancreatitis, illustrating the variability in tracer kinetics between malignant and benign lesions. In Online Resource 1, TACs and fitted curves are shown for these three patients across all time intervals. The compartment models yielding the lowest AIC for the 0–45 min interval, and with the appended late time points, are presented in Table 2. The 2T4k model had the highest percentage lesions with the lowest AIC for all scan durations. Visual inspection of the TAC fits revealed that the 1T2k model frequently failed to adequately describe the early phase of the curves. In contrast, the reversible 2T4k model more consistently captured both the early vascular component and the subsequent plateau and washout behavior. This observation was consistent with the AIC analysis, which favored the 2T4k model. Adding the 60-min timepoint increased the proportion of lesions best fitted by the 2T4k model (lowest AIC) from 62% to 73%, while the share of lesions with lowest AIC for the 2T3k model decreased from 27% to 16%. Kinetic parameters that remained significantly different after correction for multiple comparisons between malignant and benign lesions (0–60 min interval) are presented in Table 3. The kinetic results for all time intervals and compartment models (1T2K, 2T4k and 2T3k) are presented in Online Resource 2 and 3. Results from the reversible models (1T2k, and 2T4k) and from Logan plots were selected since a majority of lesions displayed reversible kinetics. For the 0–45 and 0–60 min intervals, V_T_ and V_S_ derived from the 2T4k model were significantly higher in malignant lesions, whereas k_2_ and k_4_, derived from the same model, were higher in benign lesions. The V_T_ derived from Logan plots were significantly higher in malignant lesions for all time points. In Fig. 2, boxplots of SUV_max_, TBR_max_, V_T_ (2T4k), and k_4_ (2T4k) for malignant and benign lesions are shown for the 0–60 min interval. The highest rate of parameter exclusion was observed for k_3_ and k_4_.

**Table 2:** Number of lesions with lowest AIC for each kinetic model

Models with the lowest AIC (n)					
	**0–45 min**	**0–60 min**		**0–180 min**	
1T2k	12 (11%)	11 (11%)		6 (13%)	
2T4k	64 (62%)	76 (73%)		32 (70%)	
2T3k	28 (27%)	17 (16%)		8 (17%)	
Total	104 (100%)	104 (100%)		44 (100%)

**Table 3:** Kinetic parameters and simplified methods for the 0–60 min interval differing significantly between malignant and benign lesions after Benjamini-Hochberg correction

Parameter	Model	Estimate ± SD, 95% CI	*p*-value	Marginal *R*^2^
0–60 min				
k_2_	2T4k	-0.39 ± 0.15 (-0.71 to -0.07)	0.0204	0.07
k_4_	2T4k	-0.02 ± 0.01 (-0.04 to -0.01)	0.0034	0.14
V_T_	2T4k	2.00 ± 0.43 (1.14 to 2.88)	< 0.0001	0.19
V_S_	2T4k	1.74 ± 0.38 (0.97 to 2.50)	< 0.0001	0.25
V_T_	Logan	2.12 ± 0.32 (1.48 to 2.76)	< 0.0001	0.16
SUV_max_		5.49 ± 0.74 (4.01 to 6.96)	< 0.0001	0.24
TBR_max_		3.86 ± 0.57 (2.72 to 5.00)	< 0.0001	0.20

### Comparing simplified methods to results from kinetic modelling

**Fig. 3:**
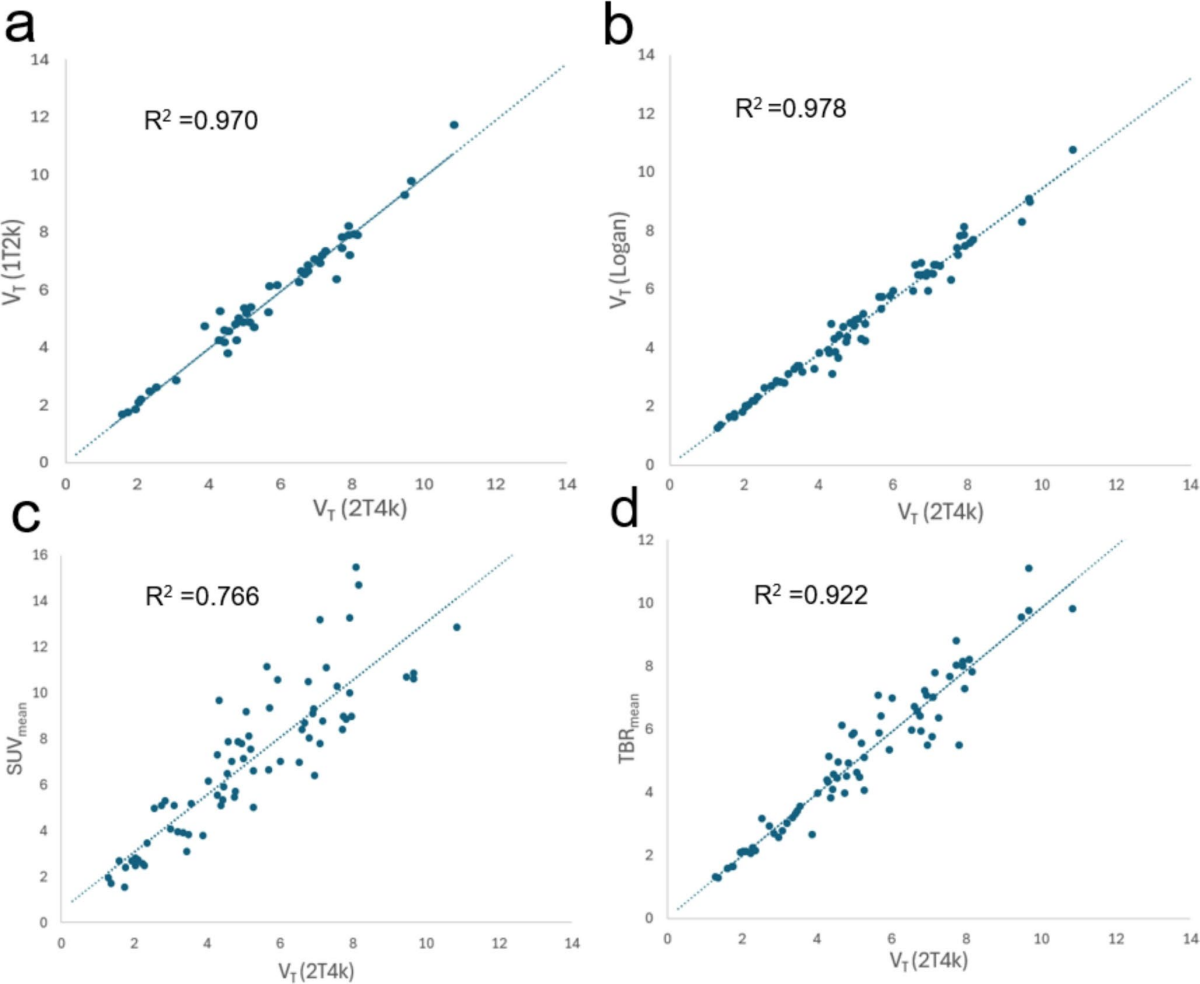
Panel (**a**) shows the correlation between V_T​_ obtained from the 1T2k- and 2T4k-models, while Panel (**b**) shows the correlation between the Logan plot and V_T_​ from the 2T4k-model. Panels (**c**) and (**d**) show scatterplots illustrating the correlations of SUV_mean​_ and TBR_mean ​_ respectively, with V_T_ ​ derived from the 2T4k model. All parameters were derived from data acquired over the 0–60 min interval

**Fig. 4:**
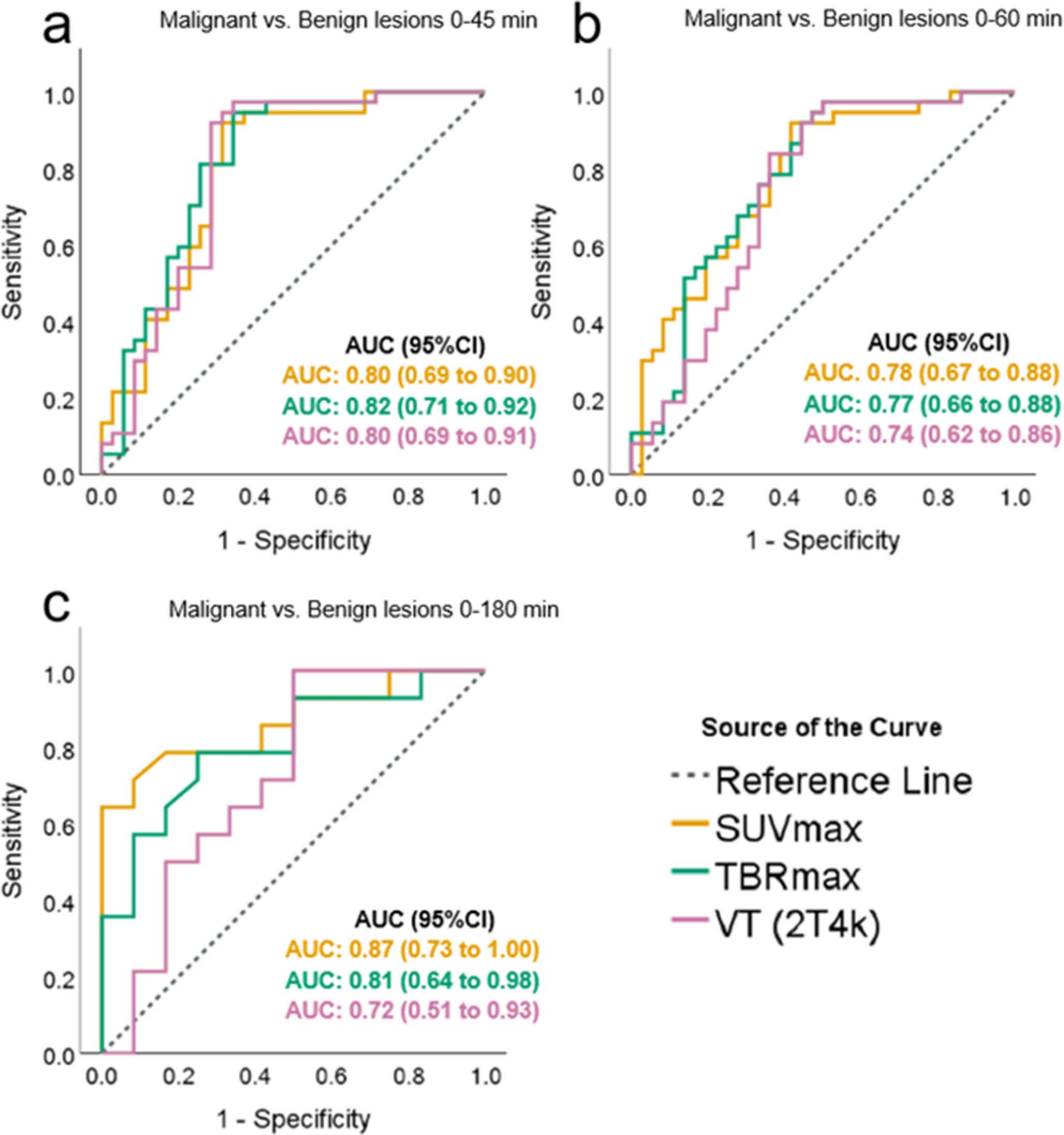
ROC curves for V_T_ derived from the 2T4k compartment model together with SUV_max_, and TBR_max_ measured in static images at the last dynamic frame (35–45 min), 60 min, and 180 min post-injection

SUV_max_, SUV_peak_, SUV_mean_, as well as TBR_max_, TBR_peak_ and TBR_mean_, were significantly higher in malignant than in benign lesions at all time points, Table 3 include the results from the linear mixed model performed on SUV_max_ and TBR_max_ for the 0–60 min interval. Results from the linear mixed model on SUV and TBR measurements from all time points are provided in Online Resource 4. Figure 2 shows boxplots of SUV_max_, TBR_max_, V_T_ (2T4k), and k_2_ (1T2k) for malignant and benign lesions. The Pearson correlation coefficients between simplified methods and V_T_ (2T4k) for the 0–60 min interval is presented in Table 4. Strong correlations were observed between SUV and V_T_ (*r* ≥ 0.784)​, and very strong correlation between TBR and V_T_ (*r* ≥ 0.902)​, for the 0–60 min interval, with strong correlations observed across all models and time points. Correlation results for the time intervals 0–45 min and 0–60 min are available in Online Resource 5. Figure 3, panels A and B, show scatterplots of V_T_ from the 1T2k versus 2T4k models and from Logan plots versus the 2T4k model, respectively. Figure 3, panels C and D, show scatterplots of SUV_mean_ and TBR_mean_ versus V_T_ (2T4k), respectively. Figure 4 shows ROC curves for SUV_max_, TBR_max_, and V_T_ (2T4k) at each of the three intervals. The area under the curve (AUC) and 95% confidence intervals are presented for each corresponding timepoint. The AUCs were comparable among V_T_ (2T4k), and SUV_max_, TBR_max_ in the 0–45 and 0–60 min intervals. At 180 min, however, SUVₘₐₓ and TBR_max_, reached a higher AUC compared with V_T_ (2T4k) derived from the 0–180 min interval.

**Table 4:** Correlation between SUV, TBR and V_T_ from the 0–60 min interval

Pearson Correlation Coefficients 95% Confidence Interval Number of Observations (*n*)			
	V_T_ (1T2k)	V_T_ (2T4k)	V_T_ (Logan)
SUV_max_	0.784 0.664–0.864 62	0.831 0.743–0.891 73	0.836 0.766–0.886 104
SUV_peak_	0.799 0.687–0.875 62	0.831 0.744–0.891 73	0.847 0.782–0.894 104
SUV_mean_	0.837 0.743–0.899 62	0.875 0.808–0.920 73	0.879 0.826–0.916 104
TBR_max_	0.902 0.842–0.940 62	0.911 0.862–0.944 73	0.920 0.884–0.945 104
TBR_peak_	0.925 0.877–0.954 62	0.928 0.888–0.955 73	0.940 0.912–0.959 104
TBR_mean_	0.958 0.930–0.974 62	0.960 0.937–0.975 73	0.964 0.947–0.975 104

## Discussion

Finding diagnostic tools that distinguish between malignant and benign lesions in patients with suspected PC is essential for accurate diagnosis, treatment planning, and avoidance of unnecessary surgery. Patients undergoing surgery due to benign disease is a well-recognized clinical concern. The primary objective of this study was to explore if kinetic modelling of dynamic [⁶⁸Ga]Ga-FAPI-46 PET can differentiate between PC and pancreatitis. Our results indicate that V_T_ derived from the 2T4k model or Logan analysis and V_S_ (2T4k) are the most promising kinetic parameters for differentiation between PC and pancreatitis. The parameter k_2_ (2T4k), reflecting radiotracer washout to blood, and k_4_ (2T4k), reflecting dissociation from specifically bound to non-specifically bound compartments were significantly lower in PC than in pancreatitis. The significantly higher V_S_ (2T4k) and V_T_ (2T4k) values in PC indicate higher FAPI binding. This may reflect elevated FAP expression in the CAFs of the tumor stroma, as well as tumor-associated factors such as increased vascular permeability and impaired tracer efflux due to the high interstitial fluid pressure characteristic of PC [36]. In a study of 17 oncology patients, imaged with [^68^Ga]Ga-FAPI-04, Chen et al. reported that parameters including K_1_, k_3_, and V_B_ may be useful for differentiating benign from malignant lesions [26]. In contrast, our findings did not indicate that k_3_ or V_B_ could differentiate between benign and malignant lesions in this cohort. However, we did find that K_1_ was significantly higher in benign lesions compared to malignant lesions for the 2T4k model at 0–45 min and 0–60 min intervals (Table 3 and Online resource 1). One possible explanation for the difference in results is that our study used a different FAPI-ligand than Chen et al., who used [^68^Ga]Ga-FAPI-04. Another reason could be that we focused on a more homogeneous cohort, composed of patients with pancreaticobiliary malignancies, chronic pancreatitis, or both lesion types concomitantly. The feasibility of using V_B_ to differentiate malignant and benign pancreatic lesions is hampered by the intersubject variability in splenic artery shape and location, thus influencing the fitted value of V_B_ [37]. Such variability may underlie the differing results observed between studies and patient populations. Several lesions showed an early TAC peak from the splenic artery, exemplified by the pancreatitis case in Fig. 1.C.

Our secondary aim was to evaluate how kinetic parameters relate to the simplified measurements in static [⁶⁸Ga]Ga-FAPI-46 PET images. Since reversible compartment models provided the best fit, the analysis focused on correlations between simplified methods (SUV and TBR) and V_T_ (2T4k). SUV showed a strong correlation with V_T_ (*r* ≥ 0.784)​, whereas TBR demonstrated a very strong correlation (*r* ≥ 0.902) for the 0–60 min interval​. The strong correlation was consistent across maximum, peak, and mean values for both SUV and TBR, and regardless of whether V_T_ was derived from the 1T2k, 2T4k, or Logan plots. These results indicate that TBR better reflect FAPI-46 receptor binding than SUV. The strong correlation between TBR_mean_ and V_T_ compared to SUV_mean_ and V_T_ has been observed in a previous study [23]. Palard-Novello et al. performed a study where ten patients with pancreaticobiliary cancer were administered [⁶⁸Ga]Ga-FAPI-46 and underwent a 90-min dynamic PET scan, in combination with arterial and venous blood sampling. They found that target-to-whole blood ratio, and SUV_mean_ at 60–70 min post injection, had a high correlation to V_T_ [27].

Furthermore, we wanted to explore whether kinetic modelling results could improve differentiation compared to SUV, and TBR. The boxplots in Fig. 2, and the results in Tables 2 and 3 show that SUV and TBR is significantly different between the two lesion types. Lower p-values, larger mean differences, and high marginal R^2^ for SUV_max_ (Table 3 and Online Resource 1) suggest that it may be the strongest discriminator between lesions. In the ROC analysis (Fig. 4), V_T_ (2T4k) was compared with SUV_max_, and TBR_max_. V_T_ (2T4k) was selected because it differed significantly between lesion types for the 0–45 and 0–60 intervals had the lowest p-values and consistently had high marginal R^2^. ROC performance was comparable between V_T_ (2T4k), SUV_max_, and TBR_max_, suggesting that kinetic modelling provided no additional discriminatory value over simplified measurements for the lesions in this cohort. These findings highlight that, although kinetic modelling provides a detailed characterization of tracer behavior, simplified metrics remain robust and clinically practical for differentiating PC from pancreatitis. Appending the late timepoints to TACs increased the fraction of lesions favoring reversible models, while simultaneously reducing the number of lesions where the irreversible model was preferred (Table 2). This suggests that inclusion of data beyond 45 min may improve the characterization of binding reversibility in a subset of lesions in FAPI-46 imaging of pancreatic cancer and pancreatitis. When comparing the number of lesions included for each parameter (Online Resource 2) it was evident that the reversible 2T4k model yielded the lowest AIC values among the tested models, indicating the best overall fit to the TACs. However, several microparameters derived from the 2T4k model, particularly k_3_ and k_4_, showed higher variability across lesions, with a relatively large proportion of estimates exceeding the predefined standard error threshold of 25%. One possible explanation is that the TACs contained limited data beyond 45 min, as the original dynamic acquisition covered only the first 45 min and at most two additional time points were appended from static scans, which may reduce the precision with which slower kinetic components such as k_3_ and k_4_ can be estimated. In addition, small residual patient motion, e.g. due to breathing, may have contributed to variability, as no explicit motion correction was applied. Visual inspection of the TAC fits demonstrated that the reversible 2T4k model more consistently captured both the early vascular component and the subsequent plateau and washout behavior.

This study has certain methodological considerations that should be acknowledged. Primarily, we did not perform blood sample analysis to determine whole-blood and plasma activity concentrations of [^68^Ga]Ga-FAPI-46. However, results from a study by Palard-Novello et al. showed that kinetic modelling results, using an IDIF without plasma to whole-blood correction correlated well with results from full kinetic modelling with plasma input functions [27]. Furthermore, we did not analyze the presence of radiometabolites. A study using [^177^Lu]Lu-FAPI-46 showed that no radiometabolites were detected even as long as 24 h after administration [38]. There remains a need to investigate the presence of radiometabolites in [^68^Ga]Ga-FAPI-46 and how they may influence kinetic modelling results. The use of newer long-axial-field-of-view PET systems with higher sensitivity and improved temporal resolution may further refine kinetic modeling accuracy in future studies. Another limitation is that the input function was derived from a VOI in the descending aorta without explicit motion or partial volume correction; therefore, minor residual effects cannot be completely excluded. Movement during the PET acquisition, spill-in effect from surrounding uptake and adjacent biliary tracer excretion through the common bile duct, when stented, could also have influenced our results.

A key strength of this study is the focus on a large homogeneous patient population, with histopathologic verification. Additionally, only kinetic parameters with a standard error below 25% were included in the analysis, ensuring that parameters with high uncertainty or potential outliers were excluded. This approach strengthens the reliability of the kinetic results. To our knowledge no test-retest study has been published for patients administered with [⁶⁸Ga]Ga-FAPI-46. Future investigations, including test-retest examinations, could help verify the reproducibility of kinetic modelling results. It would also be interesting to see more studies comparing multiple time-point imaging of [⁶⁸Ga]Ga-FAPI-46 as an alternative to single time-point static imaging in the context of improving differentiating between PC from pancreatitis [11, 16]. Our findings show that kinetic modelling offers detailed insight into tracer kinetics of pancreatic lesions, and the strong correlations between simplified methods and V_T_ suggest that they can serve as practical surrogates for distinguishing PC from pancreatitis. This suggests that simplified measures capture much of the relevant diagnostic information, highlighting their potential value for future research and study design.

## Conclusions

Kinetic modelling of [⁶⁸Ga]Ga-FAPI-46 PET demonstrates slower tracer washout and higher binding in PC compared with pancreatitis. Simplified measurements such as SUV and TBR can offer discrimination between lesion types comparable to kinetic modeling, establishing them as pragmatic alternatives for clinical application.

## Supplementary Information

Below is the link to the electronic supplementary material.

Supplementary Material 1

Supplementary Material 2

Supplementary Material 3

Supplementary Material 4

Supplementary Material 5

## Data Availability

The datasets generated during and/or analysed during the current study are available from the corresponding author on reasonable request.
