# Supplementary material for: Kinetic modelling of [⁶⁸Ga]Ga-FAPI-46 PET in pancreaticobiliary lesions: distinguishing cancer from pancreatitis
Source: Eur J Nucl Med Mol Imaging. 2026 May 6;53(9):5549–59. doi: 10.1007/s00259-026-07906-2 (PMC13314683; doi:10.1007/s00259-026-07906-2)
Supplement: Supplementary file 1 — Supplementary Material 1 [file 259_2026_7906_MOESM1_ESM.docx]

Article Title: Kinetic Modelling of [⁶⁸Ga]Ga-FAPI-46 PET in Pancreaticobiliary Lesions: Distinguishing Cancer from Pancreatitis
Journal name: European Journal of Nuclear Medicine and Molecular Imaging (EJNMMI)
Author names: Ted Nilsson, Pawel Rasinski, Ernesto Sparrelid, Antonios Tzortzakakis, Thuy A Tran, Örjan Smedby, Rimma Axelsson, Mark Lubberink, and Maria Holstensson
Corresponding author: Ted Nilsson
Affiliation: Department of Clinical Science, Intervention and Technology, Karolinska Institutet, Stockholm, Sweden and Department of Nuclear Medicine and Medical Physics, Karolinska University Hospital, Huddinge, Sweden

E-mail address: ted.nilsson@regionstockholm.se


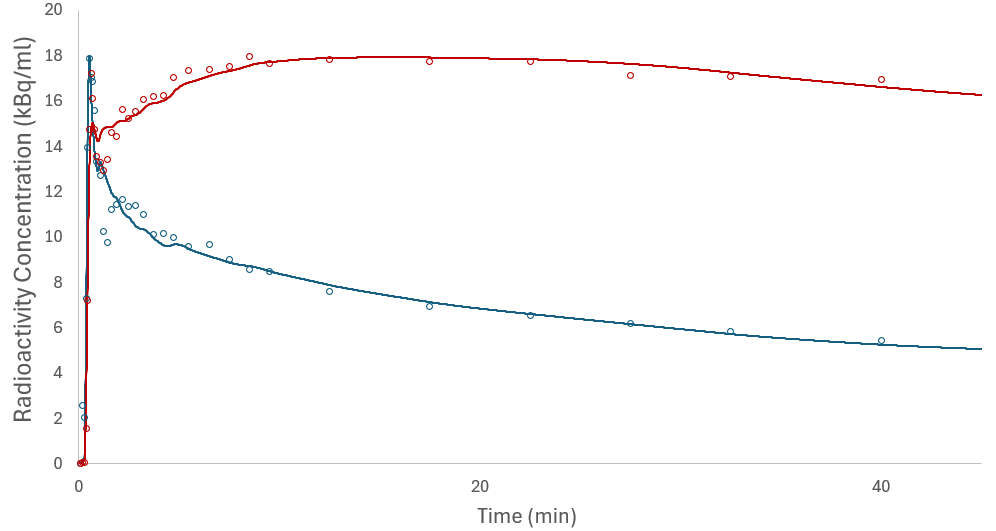


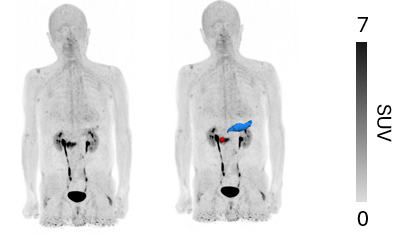


a


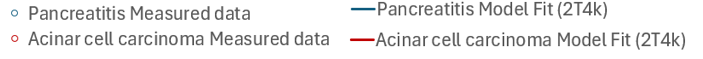


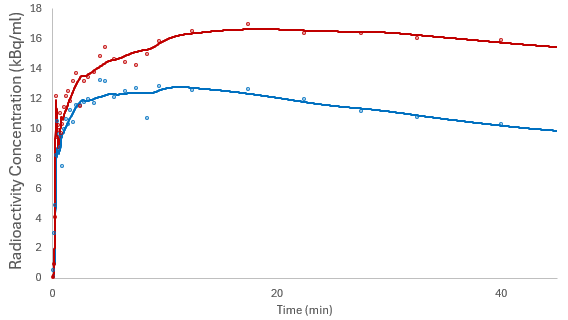


b


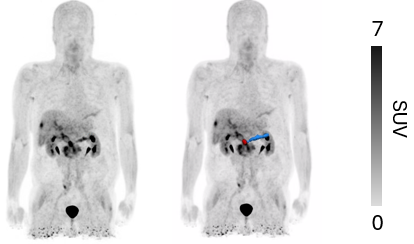


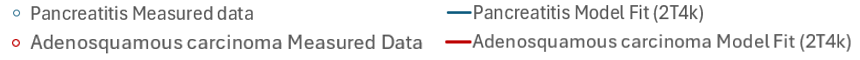


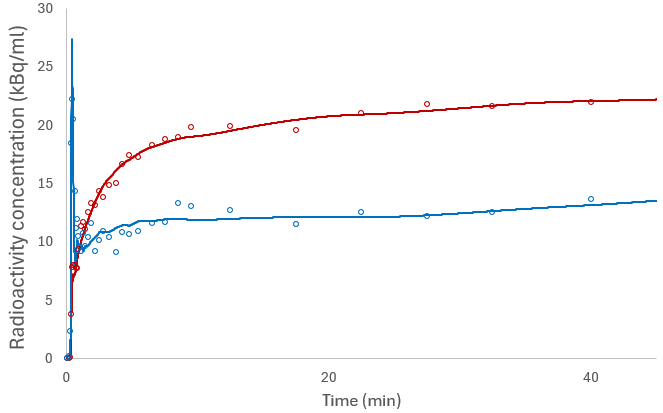


c


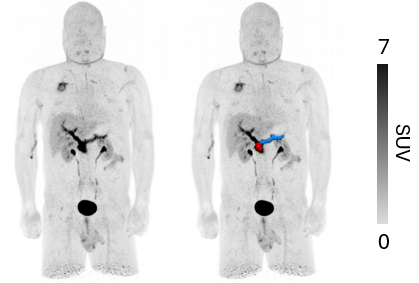


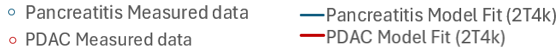


**Supplementary figure.** The TACs from 0-45 min of three patients with concomitant malignant and benign lesions of the pancreas. A) is a patient having both acinar cell carcinoma and chronic pancreatitis. B) is a patient having both adenosquamous carcinoma of the pancreas and chronic pancreatitis. C) is a patient having both Pancretatic Ductal Adenocarcinoma (PDAC) and chronic pancreatitis lesions.


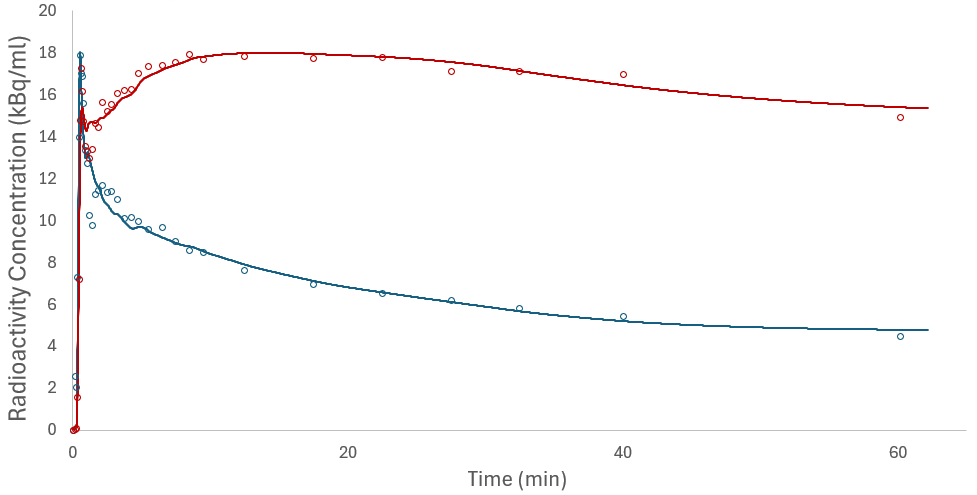


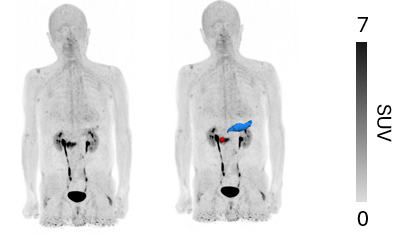


a


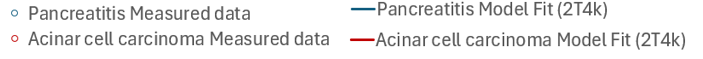


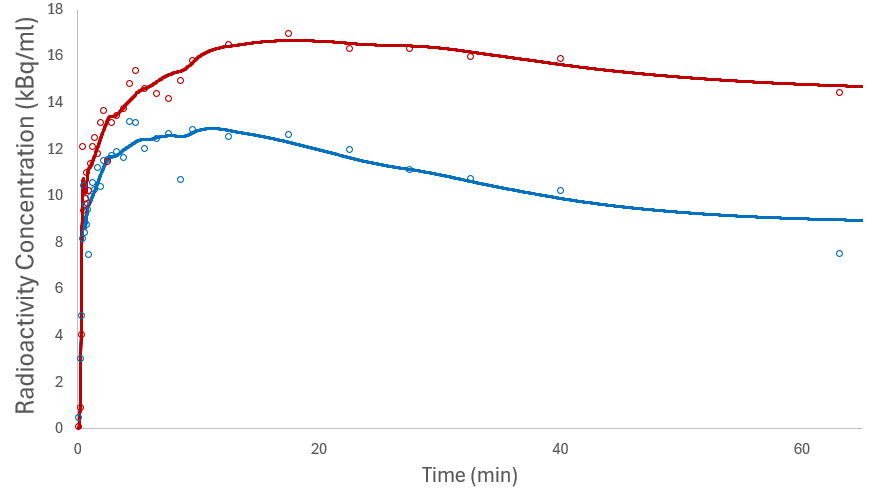


b


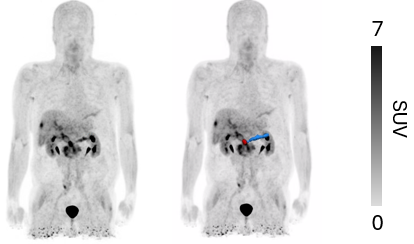


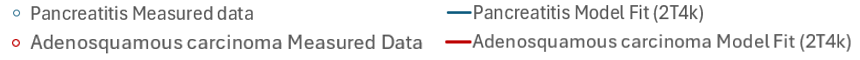


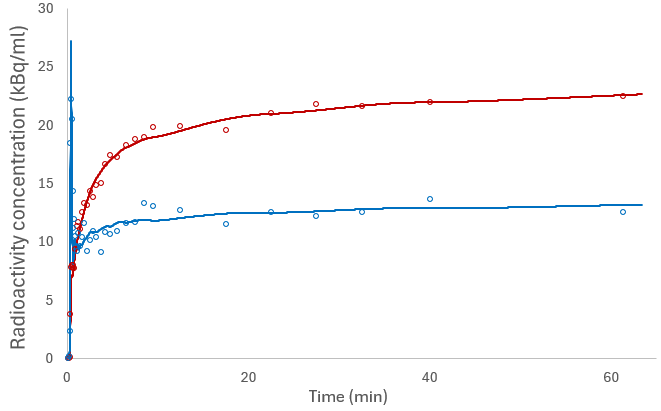


c


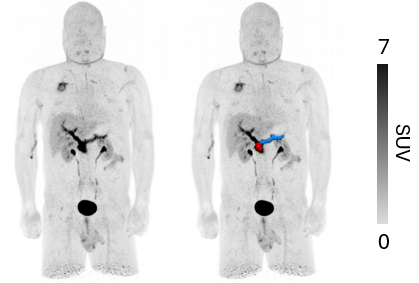


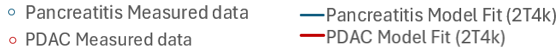


**Supllementary Figure** The TACs from 0-60 min of three patients with concomitant malignant and benign lesions of the pancreas. A) is a patient having both acinar cell carcinoma and chronic pancreatitis. B) is a patient having both adenosquamous carcinoma of the pancreas and chronic pancreatitis. C) is a patient having both Pancretatic Ductal Adenocarcinoma (PDAC) and chronic pancreatitis lesions.


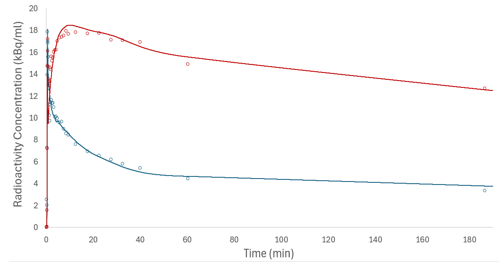


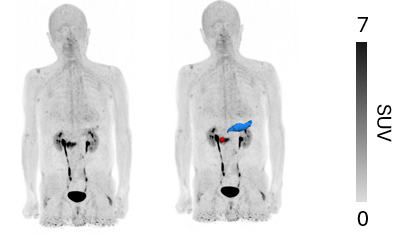


a


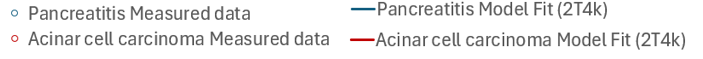


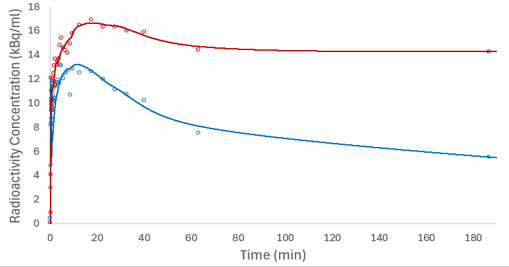


b


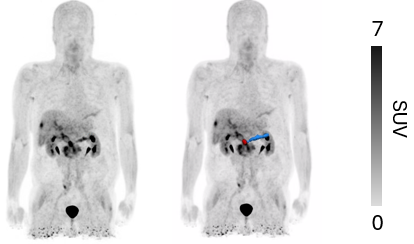


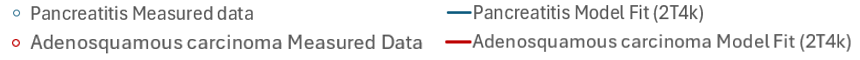


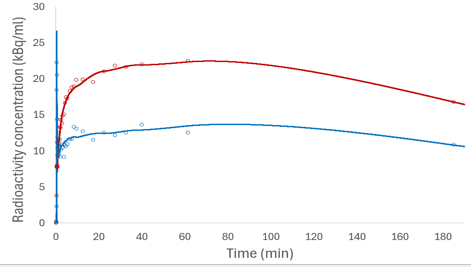


c


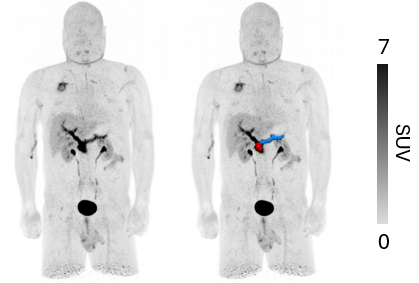


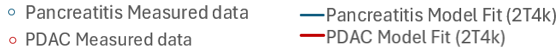


**Supplementary Figure.** The TACs from 0-180 min of three patients with concomitant malignant and benign lesions of the pancreas. A) is a patient having both acinar cell carcinoma and chronic pancreatitis. B) is a patient having both adenosquamous carcinoma of the pancreas and chronic pancreatitis. C) is a patient having both Pancretatic Ductal Adenocarcinoma (PDAC) and chronic pancreatitis lesions.
