# Supplementary material for: Kinetic modelling of [⁶⁸Ga]Ga-FAPI-46 PET in pancreaticobiliary lesions: distinguishing cancer from pancreatitis
Source: Eur J Nucl Med Mol Imaging. 2026 May 6;53(9):5549–59. doi: 10.1007/s00259-026-07906-2 (PMC13314683; doi:10.1007/s00259-026-07906-2)
Supplement: Supplementary file 2 — Supplementary Material 2 [file 259_2026_7906_MOESM2_ESM.docx]

Article Title: Kinetic Modelling of [⁶⁸Ga]Ga-FAPI-46 PET in Pancreaticobiliary Lesions: Distinguishing Cancer from Pancreatitis
Journal name: European Journal of Nuclear Medicine and Molecular Imaging (EJNMMI)
Author names: Ted Nilsson, Pawel Rasinski, Ernesto Sparrelid, Antonios Tzortzakakis, Thuy A Tran, Örjan Smedby, Rimma Axelsson, Mark Lubberink, and Maria Holstensson
Corresponding author: Ted Nilsson
Affiliation: Department of Clinical Science, Intervention and Technology, Karolinska Institutet, Stockholm, Sweden and Department of Nuclear Medicine and Medical Physics, Karolinska University Hospital, Huddinge, Sweden

E-mail address: ted.nilsson@regionstockholm.se

**Supplemental Table**. Kinetic parameters for the 0-45 min interval analysed using linear mixed models

| Parameter | Model | Estimate | p-value | Shapiro-Wilks test | p-value (after rank transformation) | Marginal R^2^ | Benjamini - Hochberg Significance |
| --- | --- | --- | --- | --- | --- | --- | --- |
| *K_1_* | 1T2k | 0.02 ± 0.02 (-0.02 to 0.07) | 0.3057 | NS | 0.2371 | 0.02 | NS |
| *k_2_* | 1T2k | -0.01 ± 0.01 (-0.02 to 0.00) | 0.0617 | S | 0.0776 | 0.04 | NS |
| *V_T_* | 1T2k | 1.27 ± 0.53 (0.18 to 2.37) | 0.0243 | S | 0.0322 | 0.05 | S |
| *vB* | 1T2k | 0.01 ± 0.01 (-0.02 to 0.03) | 0.4656 | NS | 0.6036 | 0.01 | NS |
| *K_1_* | 2T4k | -0.10 ± 0.04 (-0.19 to -0.02) | 0.0226 | S | 0.0843 | 0.05 | S |
| *k_2_* | 2T4k | -0.35 ± 0.13 (-0.61 to -0.09) | 0.0113 | S | 0.0106 | 0.04 | S |
| *k_3_* | 2T4k | -0.01 ± 0.07 (-0.17 to 0.14) | 0.8537 | S | 0.6819 | <0.01 | NS |
| *k_4_* | 2T4k | -0.04 ± 0.01 (-0.06 to -0.02) | 0.0015 | S | 0.0003 | 0.18 | S |
| *V_T_* | 2T4k | 2.23 ± 0.37 (1.47 to 2.99) | <0.0001 | NS | <0.0001 | 0.22 | S |
| *V_NS_* | 2T4k | 0.48 ± 044 (-0.43 to 1.38) | 0.2902 | S | 0.0707 | 0.01 | NS |
| *V_S_* | 2T4k | 2.07 ± 0.32 (1.41 to 2.72) | <0.0001 | NS | <0.0001 | 0.40 | S |
| *BP* | 2T4k | 1.21 ± 1.13 (-1.07 to 3.48) | 0.2912 | S | 0.0260 | 0.02 | NS |
| *vB* | 2T4k | 0.01 ± 0.01 (-0.02 to 0.04) | 0.4671 | NS | 0.5925 | 0.01 | NS |
| *V_T_* | Logan | 1.98 ± 0.31 (1.35 to 2.60) | <0.0001 | S | <0.0001 | 0.15 | - |
| *K_1_* | 2T3k | <0.01 ± 0.02 (-0.04 to 0.04) | 0.9832 | S | 0.5274 | <0.01 | NS |
| *k_2_* | 2T3k | -0.04 ± 0.02 (-0.09 to 0.00) | 0.0665 | S | 0.0668 | 0.06 | NS |
| *k_3_* | 2T3k | -0.002 ± 0.002 (-0.007 to 0.003) | 0.4239 | NS | 0.6047 | 0.01 | NS |
| *K_i_* | 2T3k | 0.01 ± 0.01 (-0.01 to 0.02) | 0.2972 | NS | 0.5786 | 0.02 | NS |
| *K_i_* | Patlak | 0.010 ± 0.004 (-0.002 to 0.017) | 0.1102 | NS | 0.1391 | 0.03 | - |

**Supplemental Table**. Kinetic parameter results for the 0-60 min interval analysed using linear mixed models

| Parameter | Model | Estimate | p-value | Shapiro-Wilks test | p-value (after rank transformation) | Marginal R^2^ | Benjamini - Hochberg Significance |
| --- | --- | --- | --- | --- | --- | --- | --- |
| *K_1_* | 1T2k | -0.01 ± 0.02 (-0.04 to 0.03) | 0.7088 | NS | 0.7203 | <0.01 | NS |
| *k_2_* | 1T2k | -0.02 ± 0.01 (-0.03 to 0.00) | 0.0314 | S | 0.0058 | 0.02 | NS |
| *V_T_* | 1T2k | 1.05 ± 0.55 (-0.06 to 2.17) | 0.0632 | S | 0.1035 | 0.03 | NS |
| *vB* | 1T2k | 0.02 ± 0.02 (-0.01 to 0.05) | 0.2475 | S | 0.4354 | 0.03 | NS |
| *K_1_* | 2T4k | -0.12 ± 0.04 (-0.21 to -0.04) | 0.0054 | S | 0.0491 | 0.07 | S |
| *k_2_* | 2T4k | -0.39 ± 0.15 (-0.71 to -0.07) | 0.0204 | S | 0.0296 | 0.04 | S |
| *k_3_* | 2T4k | -0.10 ± 0.07 (-0.24 to 0.05) | 0.1614 | NA | 0.5405 | 0.05 | NS |
| *k_4_* | 2T4k | -0.02 ± 0.01 (-0.04 to -0.01) | 0.0034 | NS | 0.0038 | 0.14 | S |
| *V_T_* | 2T4k | 2.00 ± 0.43 (1.14 to 2.88) | <0.0001 | S | <0.0001 | 0.19 | S |
| *V_NS_* | 2T4k | 0.63 ± 0.44 (-0.27 to 1.53) | 0.1593 | S | 0.0288 | <0.01 | NS |
| *V_S_* | 2T4k | 1.74 ± 0.38 (0.97 to 2.50) | <0.0001 | S | <0.0001 | 0.25 | S |
| *BP* | 2T4k | 0.45 ± 1.40 (-2.38 to 3.28) | 0.7477 | S | 0.6416 | <0.01 | NS |
| *vB* | 2T4k | -0.01 ± 0.01 (-0.03 to 0.02) | 0.6164 | NS | 0.5725 | 0.01 | NS |
| *V_T_* | Logan | 2.12 ± 0.32 (1.48 to 2.76) | <0.0001 | S | <0.0001 | 0.16 | - |
| *K_1_* | 2T3k | 0.02 ± 0.02 (-0.02 to 0.06) | 0.3505 | NS | 0.2364 | 0.01 | NS |
| *k_2_* | 2T3k | -0.02 ± 0.01 (-0.03 to 0.00) | 0.0226 | S | 0.0311 | 0.01 | NS |
| *k_3_* | 2T3k | 0.003 ± 0.001 (-0.002 to 0.007) | 0.2196 | S | 0.0438 | 0.05 | NS |
| *K_i_* | 2T3k | 0.01 ± 0.01 (0.00 to 0.02) | 0.0453 | NS | 0.0266 | 0.10 | NS |
| *K_i_* | Patlak | -0.003 ± 0.003 (-0.010 to 0.004) | 0.4017 | NS | 0.4397 | 0.01 | - |

**Supplemental Table**. Kinetic parameter results for the 0-180 min interval analysed using linear mixed models

| Parameter | Model | Estimate | p-value | Shapiro-Wilks test | p-value (after rank transformation) | Marginal R^2^ | Benjamini - Hochberg Significance |
| --- | --- | --- | --- | --- | --- | --- | --- |
| *K_1_* | 1T2k | 0.01 ± 0.05 (-0.10 to 0.09) | 0.8929 | S | 0.2357 | <0.01 | NS |
| *k_2_* | 1T2k | -0.04 ± 0.02 (-0.08 to 0.00) | 0.0381 | NS | 0.0064 | 0.16 | NS |
| *V_T_* | 1T2k | 1.85 ± 0.86 (0.09 to 3.61) | 0.0405 | S | 0.0010 | 0.26 | NS |
| *vB* | 1T2k | 0.02 ± 0.03 (-0.05 to 0.10) | 0.4732 | NS | 0.7858 | 0.04 | NS |
| *K_1_* | 2T4k | -0.04 ± 0.06 (-0.15 to 0.08) | 0.4986 | NS | 0.5469 | 0.01 | NS |
| *k_2_* | 2T4k | -0.11 ± 0.13 (-0.38 to 0.17) | 0.4379 | S | 0.5340 | 0.02 | NS |
| *k_3_* | 2T4k | -0.11 ± 0.10 (-0.3 to 0.116) | 0.2920 | S | 0.0697 | 0.12 | NS |
| *k_4_* | 2T4k | -0.02 ± 0.02 (-0.05 to 0.02) | 0.2567 | NS | 0.9157 | 0.09 | NS |
| *V_T_* | 2T4k | 1.70 ± 0.84 (-0.02 to 3.42) | 0.0531 | NS | 0.0697 | 0.15 | NS |
| *V_NS_* | 2T4k | -0.13 ± 0.84 (-1.85 to 1.60) | 0.8797 | NS | 0.9160 | <0.01 | NS |
| *V_S_* | 2T4k | 2.36 ± 0.76 (0.74 to 3.98) | 0.0073 | NS | 0.0199 | 0.39 | NS |
| *BP* | 2T4k | -0.38 ± 1.53 (-3.72 to 2.95) | 0.8058 | NS | <0.0001 | 0.01 | NS |
| *vB* | 2T4k | -0.01 ± 0.02 (-0.06 to 0.04) | 0.7297 | NS | 0.7245 | 0.01 | NS |
| *V_T_* | Logan | 1.89 ± 0.56 (0.76 to 3.02) | 0.0016 | NS | 0.0013 | 0.21 | - |
| *K_1_* | 2T3k | -0.01 ± 0.04 (-0.10 to 0.07) | 0.7895 | NS | 0.7245 | <0.01 | NS |
| *k_2_* | 2T3k | -0.04 ± 0.02 (-0.07 to -0.01) | 0.0216 | S | 0.0401 | 0.16 | NS |
| *k_3_* | 2T3k | 0.0003 ± 0.0003 (-0.0003 to 0.0010) | 0.2938 | S | 0.1317 | 0.04 | NS |
| *K_i_* | 2T3k | 0.002 ± 0.001 (0.001 to 0.005) | 0.0994 | NS | 0.0447 | 0.09 | NS |
| *K_i_* | Patlak | 0.003 ± 0.004 (-0.007 to 0.013) | 0.4508 | NS | 0.5734 | 0.15 | - |
