# Supplementary material for: Kinetic modelling of [⁶⁸Ga]Ga-FAPI-46 PET in pancreaticobiliary lesions: distinguishing cancer from pancreatitis
Source: Eur J Nucl Med Mol Imaging. 2026 May 6;53(9):5549–59. doi: 10.1007/s00259-026-07906-2 (PMC13314683; doi:10.1007/s00259-026-07906-2)
Supplement: Supplementary file 3 — Supplementary Material 3 [file 259_2026_7906_MOESM3_ESM.docx]

Article Title: Kinetic Modelling of [⁶⁸Ga]Ga-FAPI-46 PET in Pancreaticobiliary Lesions: Distinguishing Cancer from Pancreatitis
Journal name: European Journal of Nuclear Medicine and Molecular Imaging (EJNMMI)
Author names: Ted Nilsson, Pawel Rasinski, Ernesto Sparrelid, Antonios Tzortzakakis, Thuy A Tran, Örjan Smedby, Rimma Axelsson, Mark Lubberink, and Maria Holstensson
Corresponding author: Ted Nilsson
Affiliation: Department of Clinical Science, Intervention and Technology, Karolinska Institutet, Stockholm, Sweden and Department of Nuclear Medicine and Medical Physics, Karolinska University Hospital, Huddinge, Sweden

E-mail address: ted.nilsson@regionstockholm.se

**Supplemental Table**. Mean and standard deviation of each parameter included in the kinetic analysis for the 0-45 min interval

| Parameter | Model | Malignant, mean ± SD (n) | Benign, mean ± SD (n) |
| --- | --- | --- | --- |
| *K_1_* | 1T2k | 0.24 ± 0.08 (33) | 0.21 ± 0.09 (23) |
| *k_2_* | 1T2k | 0.04 ± 0.01 (33) | 0.05 ± 0.04 (23) |
| *V_T_* | 1T2k | 6.67 ± 2.25 (33) | 5.80 ± 3.34 (23) |
| *vB* | 1T2k | 0.11 ± 0.05 (15) | 0.10 ± 0.04 (17) |
| *K_1_* | 2T4k | 0.35 ± 0.15 (46) | 0.44 ± 0.27 (48) |
| *k_2_* | 2T4k | 0.44 ± 0.58 (34) | 0.77 ± 0.99 (39) |
| *k_3_* | 2T4k | 0.26 ± 0.14 (18) | 0.30 ± 0.32 (26) |
| *k_4_* | 2T4k | 0.06 ± 0.02 (27) | 0.09 ± 0.05 (26) |
| *V_T_* | 2T4k | 5.92 ± 2.05 (37) | 3.50 ± 2.20 (35) |
| *V_NS_* | 2T4k | 2.23 ± 2.13 (34) | 2.20 ± 3.06 (41) |
| *V_S_* | 2T4k | 4.15 ± 1.16 (30) | 2.07 ± 1.39 (29) |
| *BP* | 2T4k | 4.75 ± 3.89 (27) | 3.55 ± 4.19 (24) |
| *vB* | 2T4k | 0.09 ± 0.04 (14) | 0.09 ± 0.04 (22) |
| *V_T_* | Logan | 5.84 ± 2.12 (51) | 4.03 ± 2.76 (51) |
| *K_1_* | 2T3k | 0.25 ± 0.07 (38) | 0.26 ± 0.16 (26) |
| *k_2_* | 2T3k | 0.06 ± 0.03 (37) | 0.11 ± 0.14 (22) |
| *k_3_* | 2T3k | 0.02 ± 0.01 (29) | 0.02 ± 0.01 (19) |
| *K_i_* | 2T3k | 0.05 ± 0.02 (32) | 0.04 ± 0.02 (19) |
| *K_i_* | Patlak | 0.04 ± 0.02 (37) | 0.04 ± 0.02 (20) |

**Supplemental Table**. Mean and standard deviation of each parameter included in the kinetic analysis for the 0-60 min interval

| Parameter | Model | Malignant, mean ± SD (n) | Benign, mean ± SD (n) |
| --- | --- | --- | --- |
| *K_1_* | 1T2k | 0.23 ± 0.08 (33) | 0.24 ± 0.11 (29) |
| *k_2_* | 1T2k | 0.039 ± 0.01 (33) | 0.07 ± 0.08 (29) |
| *V_T_* | 1T2k | 6.55 ± 2.53 (33) | 5.60 ± 3.37 (29) |
| *vB* | 1T2k | 0.12 ± 0.06 (15) | 0.11 ± 0.04 (18) |
| *K_1_* | 2T4k | 0.33 ± 0.13 (43) | 0.45 ± 0.28 (43) |
| *k_2_* | 2T4k | 0.37 ± 0.55 (32) | 0.89 ± 1.20 (32) |
| *k_3_* | 2T4k | 0.23 ± 0.15 (13) | 0.30 ± 0.24 (23) |
| *k_4_* | 2T4k | 0.06 ± 0.03 (26) | 0.08 ± 0.03 (23) |
| *V_T_* | 2T4k | 6.04 ± 1.89 (37) | 4.21 ± 2.31 (36) |
| *V_NS_* | 2T4k | 2.56 ± 2.40 (32) | 2.41 ± 3.14 (32) |
| *V_S_* | 2T4k | 4.17 ± 1.62 (29) | 2.49 ± 1.43 (27) |
| *BP* | 2T4k | 4.93 ± 4.41 (19) | 4.47 ± 4.41 (21) |
| *vB* | 2T4k | 0.09 ± 0.04 (16) | 0.10 ± 0.04 (16) |
| *V_T_* | Logan | 5.94 ± 2.20 (51) | 4.07 ± 2.85 (53) |
| *K_1_* | 2T3k | 0.26 ± 0.12 (36) | 0.25 ± 0.14 (31) |
| *k_2_* | 2T3k | 0.06 ± 0.06 (35) | 0.09 ± 0.10 (31) |
| *k_3_* | 2T3k | 0.01 ± 0.00 (21) | 0.01 ± 0.01 (11) |
| *K_i_* | 2T3k | 0.03 ± 0.01 (23) | 0.02 ± 0.02 (16) |
| *K_i_* | Patlak | 0.03 ± 0.01 (24) | 0.03 ± 0.02 (7) |

**Supplemental Table**. Mean and standard deviation of each parameter included in the kinetic analysis for the 0-180 min interval

| Parameter | Model | Malignant, mean ± SD (n) | Benign, mean ± SD (n) |
| --- | --- | --- | --- |
| *K_1_* | 1T2k | 0.23 ± 0.10 (13) | 0.24 ± 0.14 (15) |
| *k_2_* | 1T2k | 0.04 ± 0.02 (13) | 0.08 ± 0.07 (15) |
| *V_T_* | 1T2k | 5.85 ± 1.58 (13) | 4.00 ± 2.71 (15) |
| *vB* | 1T2k | 0.16 ± 0.07 (6) | 0.13 ± 0.05 (8) |
| *K_1_* | 2T4k | 0.30 ± 0.14 (17) | 0.34 ± 0.19 (18) |
| *k_2_* | 2T4k | 0.20 ± 0.26 (13) | 0.31 ± 0.42 (15) |
| *k_3_* | 2T4k | 0.14 ± 0.16 (8) | 0.25 ± 0.08 (3) |
| *k_4_* | 2T4k | 0.05 ± 0.02 (10) | 0.07 ± 0.04 (6) |
| *V_T_* | 2T4k | 6.36 ± 1.60 (14) | 4.66 ± 2.61 (12) |
| *V_NS_* | 2T4k | 2.75 ± 1.71 (13) | 2.88 ± 2.57 (15) |
| *V_S_* | 2T4k | 4.15 ± 1.11 (10) | 1.79 ± 2.03 (7) |
| *BP* | 2T4k | 2.78 ± 2.53 (7) | 3.16 ± 3.16 (7) |
| *vB* | 2T4k | 0.10 ± 0.04 (7) | 0.11 ± 0.05 (9) |
| *V_T_* | Logan | 5.66 ± 1.41 (22) | 3.77 ± 2.26 (24) |
| *K_1_* | 2T3k | 0.23 ± 0.10 (20) | 0.24 ± 0.14 (13) |
| *k_2_* | 2T3k | 0.05 ± 0.01 (20) | 0.08 ± 0.07 (13) |
| *k_3_* | 2T3k | 0.00 ± 0.00 (20) | 0.00 ± 0.00 (13) |
| *K_i_* | 2T3k | 0.01 ± 0.00 (20) | 0.00 ± 0.00 (13) |
| *K_i_* | Patlak | 0.01 ± 0.00 (3) | 0.01 ± 0.01 (3) |
