# Supplementary material for: Kinetic modelling of [⁶⁸Ga]Ga-FAPI-46 PET in pancreaticobiliary lesions: distinguishing cancer from pancreatitis
Source: Eur J Nucl Med Mol Imaging. 2026 May 6;53(9):5549–59. doi: 10.1007/s00259-026-07906-2 (PMC13314683; doi:10.1007/s00259-026-07906-2)
Supplement: Supplementary file 4 — Supplementary Material 4 [file 259_2026_7906_MOESM4_ESM.docx]

Article Title: Kinetic Modelling of [⁶⁸Ga]Ga-FAPI-46 PET in Pancreaticobiliary Lesions: Distinguishing Cancer from Pancreatitis
Journal name: European Journal of Nuclear Medicine and Molecular Imaging (EJNMMI)
Author names: Ted Nilsson, Pawel Rasinski, Ernesto Sparrelid, Antonios Tzortzakakis, Thuy A Tran, Örjan Smedby, Rimma Axelsson, Mark Lubberink, and Maria Holstensson
Corresponding author: Ted Nilsson
Affiliation: Department of Clinical Science, Intervention and Technology, Karolinska Institutet, Stockholm, Sweden and Department of Nuclear Medicine and Medical Physics, Karolinska University Hospital, Huddinge, Sweden

E-mail address: ted.nilsson@regionstockholm.se

**Supplemental Table**. Kinetic parameters for the 0-45 min interval analysed using linear mixed models

| Parameter | Estimate | p-value | Shapiro-Wilks test | p-value (after rank transformation) | Marginal R^2^ |
| --- | --- | --- | --- | --- | --- |
| *SUV_max_* | 5.11 ± 0.73 (3.64 to 6.57) | < 0.0001 | NS | < 0.0001 | 0.20 |
| *SUV_peak_* | 4.15 ± 0.64 (2.87 to 5.44) | < 0.0001 | NS | < 0.0001 | 0.16 |
| *SUV_mean_* | 3.04 ± 0.49 (2.06 to 4.02) | < 0.0001 | NS | < 0.0001 | 0.17 |
| *TBR_max_* | 3.26 ± 0.56 (2.14 to 4.38) | < 0.0001 | S | < 0.0001 | 0.14 |
| *TBR_peak_* | 2.63 ± 0.49 (1.65 to 3.61) | < 0.0001 | S | < 0.0001 | 0.11 |
| *TBR_mean_* | 1.87 ± 0.37 (1.13 to 2.61) | < 0.0001 | S | < 0.0001 | 0.11 |

**Supplemental Table**. Kinetic parameters for the 0-60 min interval analysed using linear mixed models

| Parameter | Estimate | p-value | Shapiro-Wilks test | p-value (after rank transformation) | Marginal R^2^ |
| --- | --- | --- | --- | --- | --- |
| *SUV_max_* | 5.49 ± 0.74 (4.01 to 6.96) | < 0.0001 | NS | < 0.0001 | 0.24 |
| *SUV_peak_* | 4.43 ± 0.63 (3.17 to 5.69) | < 0.0001 | NS | < 0.0001 | 0.22 |
| *SUV_mean_* | 3.27 ± 0.46 (2.34 to 4.20) | < 0.0001 | S | < 0.0001 | 0.22 |
| *TBR_max_* | 3.86 ± 0.57 (2.72 to 5.00) | < 0.0001 | S | < 0.0001 | 0.20 |
| *TBR_peak_* | 3.09 ± 0.48 (2.12 to 4.06) | < 0.0001 | S | < 0.0001 | 0.19 |
| *TBR_mean_* | 2.25 ± 0.36 (1.53 to 2.96) | < 0.0001 | S | < 0.0001 | 0.18 |

**Supplemental Table**. Kinetic parameters for the 0-180 min interval analysed using linear mixed models

| Parameter | Estimate | p-value | Shapiro-Wilks test | p-value (after rank transformation) | Marginal R^2^ |
| --- | --- | --- | --- | --- | --- |
| *SUV_max_* | 5.68 ± 1.10 (3.47 to 7.88) | < 0.0001 | NS | < 0.0001 | 0.38 |
| *SUV_peak_* | 4.13 ± 0.93 (2.26 to 6.01) | < 0.0001 | NS | < 0.0001 | 0.31 |
| *SUV_mean_* | 2.93 ± 0.71 (1.51 to 4.35) | < 0.0001 | NS | < 0.0001 | 0.22 |
| *TBR_max_* | 5.95 ± 1.22 (3.49 to 8.40) | < 0.0001 | NS | < 0.0001 | 0.35 |
| *TBR_peak_* | 4.34 ± 0.99 (2.34 to 6.34) | 0.0001 | NS | < 0.0001 | 0.30 |
| *TBR_mean_* | 2.95 ± 0.70 (1.54 to 4.36) | 0.0001 | NS | < 0.0001 | 0.29 |

**Supplemental Table**. SUV and TBR measurements for malignant and benign lesions in the last frame of the dynamic acquisition (35-45 min).

| Parameter | Malignant, mean ± SD (n) | Benign, mean ± SD (n) |
| --- | --- | --- |
| *SUV_max_* | 14.96 ± 4.56 (51) | 10.21 ± 5.66 (53) |
| *SUV_peak_* | 12.27 ± 4.34 (51) | 8.50 ± 5.21 (53) |
| *SUV_mean_* | 9.32 ± 2.98 (51) | 6.54 ± 3.86 (53) |
| *TBR_max_* | 10.17 ± 3.81 (51) | 7.24 ± 4.57 (53) |
| *TBR_peak_* | 8.33 ± 3.57 (51) | 6.04 ± 4.25 (53) |
| *TBR_mean_* | 6.30 ± 2.31 (51) | 4.66 ± 3.20 (53) |

**Supplemental Table**. SUV and TBR measurements for malignant and benign lesions from the 60 min timepoint.

| Parameter | Malignant, mean ± SD (n) | Benign, mean ± SD (n) |
| --- | --- | --- |
| *SUV_max_* | 14.72 ± 4.24 (51) | 9.48 ± 5.53 (53) |
| *SUV_peak_* | 11.74 ± 3.82 (51) | 7.57 ± 4.67 (53) |
| *SUV_mean_* | 8.60 ± 2.64 (51) | 5.53 ± 3.53 (53) |
| *TBR_max_* | 10.66 ± 3.48 (51) | 7.07 ± 4.33 (53) |
| *TBR_peak_* | 8.47 ± 3.00 (51) | 5.63 ± 3.66 (53) |
| *TBR_mean_* | 6.19 ± 2.00 (51) | 4.15 ± 2.88 (53) |

**Supplemental Table**. SUV and TBR measurements for malignant and benign lesions from the 180 min timepoint.

| Parameter | Malignant, mean ± SD (n) | Benign, mean ± SD (n) |
| --- | --- | --- |
| *SUV_max_* | 12.93 ± 3.62 (22) | 7.25 ± 3.78 (24) |
| *SUV_peak_* | 9.87 ± 3.12 (22) | 5.74 ± 3.17 (24) |
| *SUV_mean_* | 7.03 ± 2.34 (22) | 4.10 ± 2.43 (24) |
| *TBR_max_* | 13.20 ± 3.96 (22) | 7.25 ± 4.27 (24) |
| *TBR_peak_* | 10.03 ± 3.22 (22) | 5.70 ± 3.49 (24) |
| *TBR_mean_* | 7.03± 1.92 (22) | 4.08 ± 2.72 (24) |
