# Supplementary material for: Kinetic modelling of [⁶⁸Ga]Ga-FAPI-46 PET in pancreaticobiliary lesions: distinguishing cancer from pancreatitis
Source: Eur J Nucl Med Mol Imaging. 2026 May 6;53(9):5549–59. doi: 10.1007/s00259-026-07906-2 (PMC13314683; doi:10.1007/s00259-026-07906-2)
Supplement: Supplementary file 5 — Supplementary Material 5 [file 259_2026_7906_MOESM5_ESM.docx]

Article Title: Kinetic Modelling of [⁶⁸Ga]Ga-FAPI-46 PET in Pancreaticobiliary Lesions: Distinguishing Cancer from Pancreatitis
Journal name: European Journal of Nuclear Medicine and Molecular Imaging (EJNMMI)
Author names: Ted Nilsson, Pawel Rasinski, Ernesto Sparrelid, Antonios Tzortzakakis, Thuy A Tran, Örjan Smedby, Rimma Axelsson, Mark Lubberink, and Maria Holstensson
Corresponding author: Ted Nilsson
Affiliation: Department of Clinical Science, Intervention and Technology, Karolinska Institutet, Stockholm, Sweden and Department of Nuclear Medicine and Medical Physics, Karolinska University Hospital, Huddinge, Sweden

E-mail address: ted.nilsson@regionstockholm.se

**Supplemental Table**. Correlation between SUV, TBR and V_T_ from the 0-45 min interval.

| Pearson Correlation Coefficients, 95% Confidence Interval, Number of Observations | | | |
| --- | --- | --- | --- |
|  | V_T_ (1T2k) | V_T_ (2T4k) | V_T_ (Logan) |
| SUV_max_ | 0.732  0.583–0.834 57 | 0.820  0.727–0.883  73 | 0.850  0.788–0.896 103 |
| SUV_peak_ | 0.734  0.585–0.835  57 | 0.812  0.716–0.878  73 | 0.845  0.779–0.893  103 |
| SUV_mean_ | 0.757  0.618–0.850  57 | 0.821  0.729–0.884  73 | 0.859  0.799–0.903  103 |
| TBRmax | 0.925  0.875–0.955  57 | 0.955  0.929–0.972  73 | 0.962  0.945–0.974  103 |
| TBRpeak | 0.902  0.838–0.941  57 | 0.952  0.924–0.969  73 | 0.945  0.920–0.963  103 |
| TBRmean | 0.955  0.925–0.974  57 | 0.962  0.941–0.976  73 | 0.977  0.966–0.984  103 |

**Supplemental Table**. Correlation between SUV, TBR and V_T_ from the 0-60 min interval.

| Pearson Correlation Coefficients, 95% Confidence Interval, Number of Observations | | | |
| --- | --- | --- | --- |
|  | V_T_ (1T2k) | V_T_ (2T4k) | V_T_ (Logan) |
| SUV_max_ | 0.784  0.664–0.864 62 | 0.831  0.743–0.891  73 | 0.836  0.766–0.886 104 |
| SUV_peak_ | 0.799  0.687–0.875  62 | 0.831  0.744–0.891  73 | 0.847  0.782–0.894  104 |
| SUV_mean_ | 0.837  0.743–0.899  62 | 0.875  0.808–0.920  73 | 0.879  0.826–0.916  104 |
| TBRmax | 0.902  0.842–0.940  62 | 0.911  0.862–0.944  73 | 0.920  0.884–0.945  104 |
| TBRpeak | 0.925  0.877–0.954  62 | 0.928  0.888–0.955  73 | 0.940  0.912–0.959  104 |
| TBRmean | 0.958  0.930–0.974  62 | 0.960  0.937–0.975  73 | 0.964  0.947–0.975  104 |

**Supplemental Table**. Correlation between SUV, TBR and V_T_ from the 0-180 min interval.

| Pearson Correlation Coefficients 95% Confidence Interval Number of Observations | | | |
| --- | --- | --- | --- |
|  | V_T_ (1T2k) | V_T_ (2T4k) | V_T_ (Logan) |
| SUV_max_ | 0.829  0.661–0.918 28 | 0.781  0.564–0.897  26 | 0.864  0.765–0.923 46 |
| SUV_peak_ | 0.849  0.697–0.928  28 | 0.791  0.582–0.902  26 | 0.875  0.784–0.929  46 |
| SUV_mean_ | 0.893  0.780–0.950  28 | 0.859  0.706–0.935  26 | 0.914  0.848–0.952  46 |
| TBRmax | 0.829  0.660–0.918  28 | 0.799  0.596–0.906  26 | 0.836  0.721–0.906  46 |
| TBRpeak | 0.881  0.756–0.944  28 | 0.820  0.634–0.916  26 | 0.867  0.770–0.924  46 |
| TBRmean | 0.967  0.930–0.985  28 | 0.942  0.873–0.974  26 | 0.955  0.920–0.975  46 |
